# The Effect of a Flexible Electrode on the Electro Deformability of an Actuating Unit of a MDI-Polyurethane Composite Fiber Membrane Filled with BaTiO_3_

**DOI:** 10.3390/membranes12090878

**Published:** 2022-09-12

**Authors:** Gang Lu, Changgeng Shuai, Yinsong Liu, Xue Yang, Xiaoyang Hu

**Affiliations:** 1 Institude of Noise and Vibration, Naval University of Engineering, Wuhan 430033, China; 2 Key Laboratory of Ship Vibration and Noise, Wuhan 430033, China

**Keywords:** polyurethane dielectric elastomer (PUDE), flexible electrode, electro deformability, polyurethane composite fiber membrane, actuating unit

## Abstract

The electro deformability of an actuating unit of a polyurethane dielectric elastomer (PUDE) is affected by many factors. The agglomeration of dielectric fillers faced by the traditional dielectric modification methods will lead to the instability of the actuation performance of dielectric composites. In addition, the electro deformability (ability of deformation after voltage loading) is great affected by the selection of flexible electrodes and packaging technology. Based on the research findings, Diphenylmethane-4,4′-diisocyanat (MDI)-polyurethane dielectric composite fiber membrane filled with barium titanate (BaTiO_3_) is prepared using coaxial spinning, and this study then analyzes the effects of the types of flexible electrodes and coating methods on the electro deformability of the actuating unit of the dielectric composite fiber membrane. It is found that the electro deformability of the actuating unit coated with the single-walled carbon nanotube (SWNT) flexible electrode is better than that of the perfluoropolyether conductive grease (PCG) or the traditional conductive carbon grease (CCG) electrode in various degrees. When the loading voltage is 20 kV, the electro deformability of the actuating unit coated with SWNT flexible electrode exceeds the latter two electrodes by 13.8%; when the SWNT flexible electrode is encapsulated by physical surface implantation (PSI), the electric deformation of the actuating unit is higher than that of the solvent suspension dispersion (SSD).

## 1. Introduction

As one of the popular research topics in dielectric elastomer smart materials, polyurethane dielectric elastomer (PUDE) has widely application in the designs of flexible actuating units and underwater bionic machinery [1] because of its extremely fast response speed, high electromechanical conversion efficiency, excellent designability, and high environmental tolerance at the molecular level [2]. Thermoplastic polyurethane (TPU) can lead to the occurrence of physical cross-linking owing to intermolecular hydrogen bonding. When heated, this material can transform into liquid before returning to an elastomer state because of cooling. This diversifies the method of TPU processing while endowing PUDE with the massive potential for development [3].

**Figure 1:**
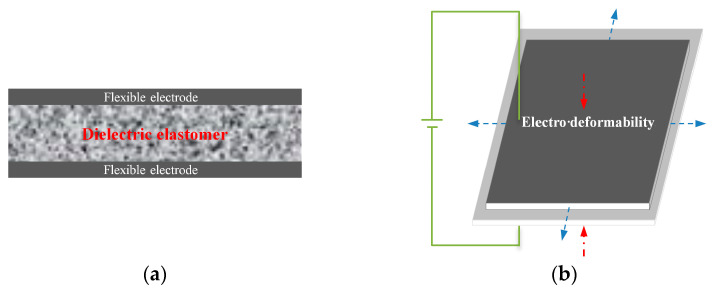
Schematic diagram of the DE actuating unit and its electro deformability. (**a**) Dielectric elastomer actuator, (**b**) Principle of electro deformability.

The PUDE actuating unit is generally composed of flexible electrodes and a PUDE. Its actuating unit is like a “sandwich” structure, which will extend in the plane under the external voltage load and then achieve the target actuating effect based on the intelligent structure design. Its principle is shown in Figure 1.

Starting from increasing the interface effect of dielectric composite materials, Chen Tian [4] prepared a PUDE actuator by modifying titanium dioxide on graphene and compounding it with PUDE. The research shows that the interface effect of modified polyurethane dielectric elastomer improved significantly, and the dielectric property and electric deformation ability also greatly improved. As everyone knows, it is a universal and effective way to enhance the mechanical and electrical properties of polymer composites by physical blending of ceramic particles with high dielectric properties. Among many ceramic particles, BaTiO_3_ is a typical ceramic particle that has good dielectric properties and has been widely used in inexpensive electronic devices [5] The electro deformability of a PUDE actuating unit is affected by many factors, such as the PUDE’s own properties and the pre stretching rate, flexible electrode, etc. It is mainly related to the dielectric or electromechanical sensitivity of the matrix material and the packaging of the actuating unit. The dielectric or electromechanical sensitivity factors are closely correlated with the orientation polarization [6] of the PUDE matrix materials and the interface polarization [7] between the dielectric fillers. On this basis, the dielectric or electromechanical sensitivity factor is inversely proportional to the elastic modulus of the modified dielectric composites [8]. On the other hand, the packaging process of the actuating unit involves the coating of flexible electrodes [9] and the pre tensile state of dielectric materials [10].

Despite the above, most of the conventional preparation methods based on PUDE composites focus on physical blending of all components [11], although there are many multi-factor matters faced by the traditional dielectric modification, such as the agglomeration of dielectric filler in the matrix material [12] and the contradiction between the dielectric constant and modulus [13] of the dielectric composite materials. In consideration of this, the polyurethane composite fiber membrane is prepared in this study using coaxial electrospinning [14], so that the dielectric filler BaTiO_3_ can fill in polyurethane fiber in a fixed direction, which was used as a matrix material of the actuating unit. Next, this study analyzes the effects of the types of flexible electrodes and coating methods on the electro deformability of polyurethane composite fiber membrane actuating unit.

## 2. Experimental Techniques

### 2.1. The Main Raw Materials

As shown in Table 1, the main chemical materials used in this study included polyether MDI polyurethane prepolymer (MDI-PUP) with isocyanate group (NCO) content 13.25–13.65 wt.%; polyether polyol (PA), chemical purity with relative molecular weight (i.e., 2000); butanediol (BDO), analytical purity. According to the literature, the types of flexible electrodes commonly used in the research of dielectric elastomers are CCG, PCG, SWNTs, and other electrodes. Here, the above three as the main research objects [15].

### 2.2. The Main Devices

Main devices is shown in Table 2, the electrospinning device is mainly used for the formation of fiber membranes. The formation state of fiber membranes is controlled by controlling the voltage parameters, environmental parameters, size of the spinning head, distance between the spinning head and the collecting device, rotation speed of the collecting roller, and other parameters that affect the morphology of fiber membranes.

### 2.3. Experimental Procedure

(1) Preparation of polyurethane composite fiber membrane filled with BaTiO_3_

**Figure 2:**
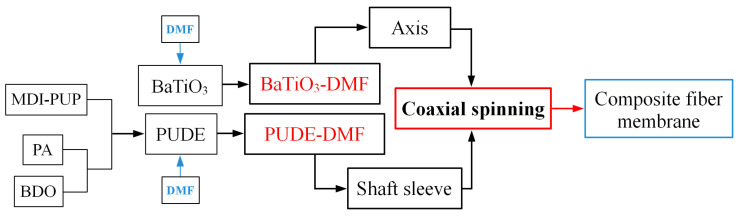
Flow chart for preparation of polyurethane composite fiber membrane.

**Figure 3:**
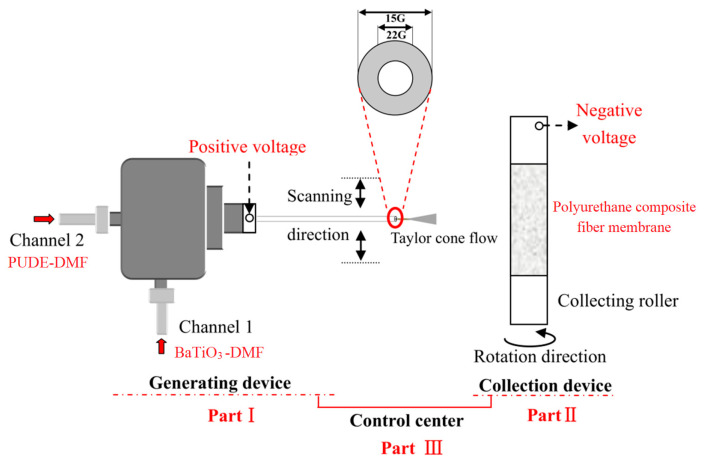
Schematic diagram of the coaxial spinning technique.

As shown in Figure 2, the PUDE is prepared by mixing at 70 °C in the proportions of MDI-PUP, PA, and BDO of 100, 30, and 12.8, respectively. Then, the metered PUDE and BaTiO_3_ are dissolved or mixed with DMF to prepare the PUDE solution and BaTiO_3_ suspension (the mass ratio of PUDE:DMF of 1:5, the mass ratio of BaTiO_3_:DMF of 1:1000/2:1000/3:1000). Next, the above two solutions are loaded into the shaft sleeve and shaft channel of the coaxial spinning device, respectively. Four kinds of polyurethane composite fiber membranes with the mass ratio of PUDE:BaTiO_3_ of 100:0, 100:0.5, 100:1.0, and 100:1.5 were obtained by adjusting the coaxial spinning parameters. The principles of the coaxial spinning technology are shown in Figure 3.

As shown in Figure 3, the coaxial spinning device is composed of three parts: fiber generation, fiber membrane collection, and control. Specifically, the liquid ejected from the spinning head will form a Taylor cone flow under the action of high voltage that will be collected on the collection roller. During that time, the morphology of the composite fiber membrane and the filling state of BaTiO_3_ can be controlled by adjusting the voltage, the scanning speed, the ejection speed, the speed of the collection roller, and the environmental factors (temperature and humidity) of the device. In this way, the polyurethane composite fiber membrane with the target morphology can be obtained. After adjustment, the main coaxial spinning parameters are as follows: spinning solution concentration 20%, pushing speed 0.7 mL/h, voltage 12.5 kV, spinning head size 22 G, humidity no more than 25%, temperature no more than 55 °C.

(2) Packaging of polyurethane composite fiber membrane actuating unit

The packaging of polyurethane composite fiber membrane actuating units mainly involves the selection of a flexible electrode, coating method, and fiber membrane pre stretching rate. This study focuses on the selection of the flexible electrode and the coating method only. The pre stretching ratio is set to 2, and the circular insulating epoxy resin (2 mm) is selected as the constraint frame.

Specifically, three kinds of flexible electrode are selected: single-walled carbon nanotubes (SWNTs), perfluoropolyether conductive grease (PCG), and conductive carbon grease (CCG). Among them, the SWNTs are coated using physical surface implantation (PSI) and solvent suspension dispersion (SSD). The former method involves arranging and stacking the single-walled carbon nanotubes on the surface of the dielectric fiber membrane to form a flexible electrode. The latter involves dispersing the carbon nanotubes in the ether solution through ultrasonic oscillation and then coating the suspension on the surface of the fiber membrane to form an electrode under the volatilization of the solvent. Additionally, the other two electrodes can be coated directly with cotton swabs.

Specifically, the fiber film is pre stretched and constrained by epoxy frame; then, flexible electrodes are coated on the upper and lower surfaces of the film, and finally, copper foil is drawn out. The electrode mass used on the upper and lower surfaces of the film is 20 mg, and the diameter of the coating surface is 10 mm.

### 2.4. Testing Methods

(1) Micro morphology characterization

The surface morphology of the fiber membrane and the arrangement of BaTiO_3_ in the fiber bundle are observed by the electron microscope. The equipment information is shown in Table 3.

(2) Electro deformability test

The first step before the electrical deformation test is to test the dielectric sensitivity factor. Considering that the dielectric sensitivity factor β is expressed in Equation (1), the dielectric constant and elastic modulus are tested as follows.

(1) $$\beta =\frac{\varepsilon_{r}}{Y}$$

It can be seen from this equation that for the dielectric material, the greater the dielectric constant and the lower the elastic modulus, the higher the dielectric sensitivity. Therefore, it is essential to balance the elastic modulus of materials while improving the dielectric properties of materials.

The dielectric properties of the samples are measured using the dielectric constant tester of model 6632-1s from the Teng Skye company. The test frequency ranges from 10 Hz to 500 Hz, the test temperature is room temperature, and the sample size is 10 mm.

Following the national standard GB/T 528-1998, the sample is cut into multiple 2 mm × 5 mm × 2 mm dumbbell-shaped splines at a tensile rate of 200 mm/min. The elastic modulus is calculated based on the slope of the initial part (deformation less than 5%) on the stress–strain curve. It is necessary to take the median value of five parallel test values [16].

**Figure 4:**
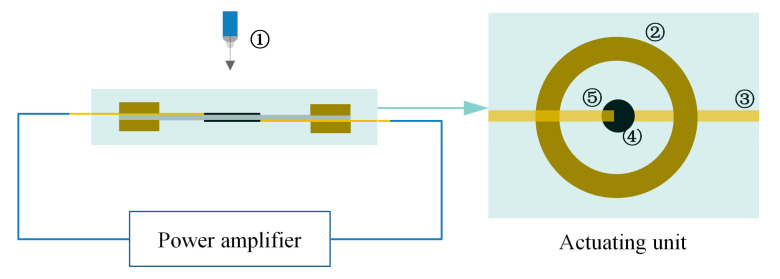
Schematic diagram of electrostrain experimental platform. (1) High-speed camera, (2) Fixed frame, (3) Copper foil, (4) Flexible electrode, (5) Polyurethane composite fiber membrane.

The next step is to verify the dielectric sensitivity factor, i.e., the test of electro deformation. The upper and lower surfaces of the flexible electrodes are led out with copper foil, and then the outgoing line is connected to the power amplifier; the machine information is as follows: RK2674A/DC 10 mA, the maximum output voltage is 20 kV, Shenzhen meirike Electronic Technology Co., Ltd. (Shenzhen, China). In addition, the focus of the high-speed camera is aligned with the flexible electrode covering part. Finally, the output voltage is adjusted through the power amplifier, and the camera can collect the electro deformability of the flexible electrode under the voltage. The electro deformability rate (*S_A_*) of the polyurethane fiber membrane actuating unit can be obtained through data processing, as shown in Equation (2). The test platform is shown in Figure 4.

(2) $$S_{A}=\frac{S_{2}-S_{1}}{S_{1}}\times 100\%$$

where *S*_1_ and *S*_2_ represent the area of the coated electrode area before and after deformation of the actuating unit, respectively.

## 3. Experimental Results

### 3.1. Micro Morphology of Fiber Membrane and Arrangement State of BaTiO_3_

**Figure 5:**
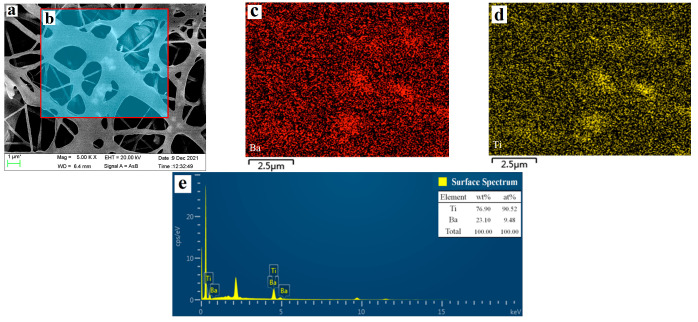
SEM & EDS analysis of polyurethane composite fiber membrane. Reprinted/adapted with permission from Ref. [16]. 2022, Membranes. (**a**) Electron microscope picture, (**b**) Energy spectrum analysis of selected areas in (**a**), (**c**) Distribution of Ba element, (**d**) Distribution of Ti element, (**e**) Content ratio of Ba element and Ti element.

Figure 5a shows the micro morphology of polyurethane fiber network. The white spots (Particle size less than 1μm) in the fiber bundle are BaTiO_3_ injected by coaxial spinning, which suggests that the BaTiO_3_ is dispersed in the polyurethane fiber. In addition, region b in Figure 5a is selected for energy spectrum analysis to explain the distribution of BaTiO_3_ in the overall fiber membrane in detail, based on which the Figure 5c,d are obtained. Figure 5c,d reveal that Ba and Ti are evenly dispersed in the selected region, which further indicates that the BaTiO_3_ shows good dispersion in polyurethane fiber membrane, keeping in line with the expectation. Figure 5e shows the content of Ba and Ti elements.

### 3.2. Electro Deformability of the Fiber Membrane Actuating Unit

(1) Effect of flexible electrode on electro deformability of fiber membrane

**Figure 6:**
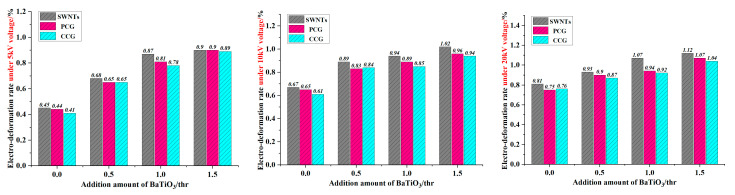
Diagram for electro deformability of actuating unit encapsulated with different electrodes.

Table 4 lists the dielectric properties and dielectric sensitivity factors of polyurethane composite fiber membranes based on different parts of BaTiO_3_ filled. Table 5 shows the electro deformability data for the actuating unit encapsulated with different electrodes. Based on the data in Table 5, Figure 6 is drawn to compare the differences in electro deformability of different actuating units.

Figure 6 shows that the electro deformability of an actuating unit of the encapsulated SWNT electrode is generally better than that of PCG and CCG under all three loading voltages. The SWNTs coated on the surface of the polyurethane material formed a three-dimensional reinforced conductive network that brought uniform and permanent conductivity to the material with little impact on other properties of the material [17]. When the amount of BaTiO_3_ added is 1 phr, the electro deformability of the actuating unit encapsulated with SWNTs electrode exceeds that encapsulated with the other two electrodes by more than 13.8% at 20 kV. When the loading voltage is increased, the electro-induced deformation of the actuator unit encapsulated with SWNTs tended to increase compared with the other two electrodes.

(2) Effect of coating methods of SWNTs electrode on electro deformability of the actuating unit

**Figure 7:**
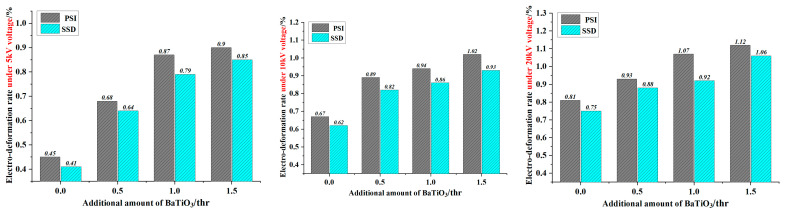
Diagram for electro deformability of the actuating unit under different coating methods.

Table 6 lists the electro deformability data for the SWNT electrode actuating units based on different coating methods. Figure 7 is given based on the data in Table 6 to clearly compare the performance of various actuating units.

**Figure 8:**
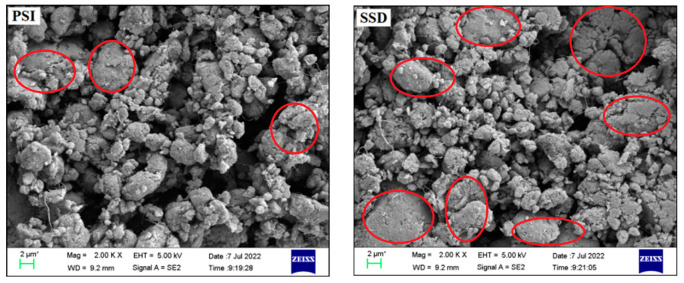
SEM micrograph of flexible electrode of PSI and SSD coating methods. The red circle means that the single-walled carbon nanotube electrode is agglomerated here.

As demonstrated in Figure 7, the electro deformability of the actuating unit prepared by PSI electrode coating is obviously better than that coated with SSD. This is related to the better dispersion of the SWNTs obtained by PSI, and this result can be seen in Figure 8, in which the scanning electron microscope images show several particularly serious agglomerations in the area separately circled in the right image; in contrast, the carbon nanotubes in the left image are evenly distributed. Meanwhile, the flexible electrode obtained by this method is cleaner, with adjustable direction and thickness. In this way, when the voltage is loaded, the synergistic effects of the electrode and the dielectric fiber membrane become stronger, and electro deformability will be better. Obviously, these results cannot be achieved by SSD.

## 4. Conclusions

(1) For the polyurethane dielectric composite fiber membrane of this system, the electro deformability of the actuating unit encapsulated with the SWNT flexible electrode is higher than that coated with PCG and CCG electrode. When the loading voltage is 20 kV, the performance of the SWNT electrode is better by 13.8% than that of PCG and CCG.
(2) For the flexible electrode based on SWNTs coating, the electro deformability of the actuating unit is better using the PSI method than that of the SSD.
(3) In the future, the influence of the types of carbon nanotubes on the electro deformation ability of the fiber membrane materials in this system should be studied. In addition, it will be very meaningful to explore the microscopic synergistic effect between carbon nanotubes and fiber membrane and the change law of electrodes when voltage is applied.

In conclusion, SWNTs can serve as a flexible electrode with reduced coating cost and improved coating technology for intelligent actuating units. In addition, the research on dielectric elastomer involves a wide range of fields, and the academic community should consider systematizing the mature test technologies such as dielectric elastomer molding and unit packaging, which will be beneficial to the early realization of engineering of this promising technology.

## Figures and Tables

**Table 1:** The main raw materials.

Items	Manufactor
BaTiO_3_ (Particle size less than 1 μm)	China (Shanghai) Macklin Biochemcal Co., Ltd.
Polyether Diphenylmethane-4,4′-diisocyanate (MDI) polyurethane prepolymer (MDI-PUP)	China (Dongguan) polymerized rubber productsCo., Ltd.
Polyether polyol additive (PA)	China (Dongxu) Industry Manufacturing Co., Ltd.
1,4-butanediol (BDO)	China (Wuhan) xiangjiutong Biotechnology Co., Ltd.
Single-walled carbon nanotubes (SWNTs)	China (Chengdu) Zhongke Shidai Naneng Co., Ltd.
Perfluoropolyether conductive grease (PCG)	China (Shenzhen) Songsen New Material Technology Co., Ltd.
Carbon conductive grease (CCG)	China (Shenzhen) Yuanzhuang Electronics Co., Ltd.
N. N-dimethylformamide (DMF)	China (Wuhan) xiangjiutong Biotechnology Co., Ltd.
Ether (ET)	China (Wuhan) xiangjiutong Biotechnology Co., Ltd.

**Table 2:** The main devices.

Instrument	Model	Manufactor
Vacuum drying oven	DZF-6050AB	China (Shanghai) Jingqi Co., Ltd.
Electrospinning apparatus	LT-Pro	China (Shenzhen) Tongli micro nano Co., Ltd.

**Table 3:** Electron microscope information.

Device	Model	Remarks
Scanning Electron Microscope	Zeiss Merlin	The signal sources were backscattered signal and secondary electron signal; the accelerating voltage was 20 kV.

**Table 4:** Dielectric properties and dielectric sensitivity factors of MDI-polyurethane composite fiber membranes.

Samples	10 Hz		100 Hz		Y/MPa	Dielectric Sensitivity Factor	
	ε_1_″	Tanα_1_	ε_2_″	Tanα_2_		β_10Hz_	β_100Hz_
DEM-BaTiO_3_/Blank	14.362	0.198	4.905	0.097	2.15	6.68	2.28
DEM-BaTiO_3_/0.5 thr	18.037	0.157	5.583	0.126	2.31	7.81	2.42
DEM-BaTiO_3_/1.0 thr	26.623	0.263	9.964	0.145	2.65	10.05	3.76
DEM-BaTiO_3_/1.5 thr	41.915	0.416	12.125	0.109	3.18	13.18	3.81

**Table 5:** Electro deformability data of actuating unit encapsulated with different electrodes.

Items	5 kV/%			10 kV/%			20 kV/%		
	SWNTs	PCG	CCG	SWNTs	PCG	CCG	SWNTs	PCG	CCG
DEM-BaTiO_3_/Blank	0.45	0.44	0.41	0.67	0.65	0.61	0.81	0.75	0.76
DEM-BaTiO_3_/0.5 thr	0.68	0.65	0.65	0.89	0.83	0.84	0.93	0.90	0.87
DEM-BaTiO_3_/1.0 thr	0.87	0.81	0.78	0.94	0.89	0.85	1.07	0.94	0.92
DEM-BaTiO_3_/1.5 thr	0.90	0.90	0.89	1.02	0.96	0.94	1.12	1.07	1.04

DEM-BaTiO_3_/X thr: Dielectric elastomer fiber membrane filled with X thr BaTiO_3_; SWNTs—Samples coated with SWNTs electrodes, PCG—Samples coated with PCG electrodes, CCG—Samples coated with CCG electrodes.

**Table 6:** Electro deformability data for the actuating unit under different electrode coating methods.

Items	5 kV/%		10 kV/%		20 kV/%	
	PSI	SSD	PSI	SSD	PSI	SSD
DEM-BaTiO_3_/Blank	0.45	0.41	0.67	0.62	0.81	0.75
DEM-BaTiO_3_/0.5 thr	0.68	0.64	0.89	0.82	0.93	0.88
DEM-BaTiO_3_/1.0 thr	0.87	0.79	0.94	0.86	1.07	0.92
DEM-BaTiO_3_/1.5 thr	0.90	0.85	1.02	0.93	1.12	1.06

## Data Availability

The data presented in this study are available on request from the corresponding author.
